# A CPO-Optimized Enhanced Linear Active Disturbance Rejection Control for Rotor Vibration Suppression in Magnetic Bearing Systems

**DOI:** 10.3390/s26020456

**Published:** 2026-01-09

**Authors:** Ting Li, Jie Wen, Tianyi Ma, Nan Wei, Yanping Du, Huijuan Bai

**Affiliations:** College of Mechanic and Electronic Engineering, Beijing Institute of Graphic Communication, Beijing 102600, China; liting@bigc.edu.cn (T.L.);

**Keywords:** active magnetic bearings, crested porcupine optimizer, enhanced linear active disturbance rejection control, linear extended state observer

## Abstract

To mitigate rotor vibrations in magnetic bearing systems arising from mass imbalance, this study proposes a novel suppression strategy that integrates the crested porcupine optimizer (CPO) with an enhanced linear active disturbance rejection control (ELADRC) framework. The approach introduces a disturbance estimation and compensation scheme based on a linear extended state observer (LESO), wherein both the LESO bandwidth $\omega_{0}$ and the LADRC controller parameter $\omega_{c}$ are adaptively tuned using the CPO algorithm to enable decoupled control and real-time disturbance rejection in complex multi-degree-of-freedom (DOF) systems. Drawing inspiration from the crested porcupine’s layered defensive behavior, the CPO algorithm constructs a state-space model incorporating rotor displacement, rotational speed, and control current, while leveraging a reward function that balances vibration suppression performance against control energy consumption. The optimized parameters guide a real-time LESO-based compensation model, achieving accurate disturbance cancelation via amplitude-phase coordination between the generated electromagnetic force and the total disturbance. Concurrently, the LADRC feedback structure adjusts the system’s stiffness and damping matrices to improve closed-loop robustness under time-varying operating conditions. Simulation studies over a wide speed range (0~45,000 rpm) reveal that the proposed CPO-ELADRC scheme significantly outperforms conventional control methods: it shortens regulation time by 66.7% and reduces peak displacement by 86.8% under step disturbances, while achieving a 79.8% improvement in adjustment speed and an 86.4% reduction in peak control current under sinusoidal excitation. Overall, the strategy offers enhanced vibration attenuation, prevents current saturation, and improves dynamic stability across diverse operating scenarios.

## 1. Introduction

Active magnetic bearings (AMBs), characterized by their non-contact operation, lack of lubrication requirements, low acoustic noise, and high controllability [1], are increasingly utilized in systems where precision and efficiency are critical. These include high-speed rotating machinery, flywheel-based energy storage units, and turbo-compressor applications [2,3,4,5]. However, due to manufacturing imperfections, rotors often suffer from unavoidable mass imbalance, which tends to generate synchronous vibrations. These vibrations, in turn, can compromise system stability and degrade control performance, occasionally leading to severe issues such as rotor–stator rubbing. As such, developing robust and efficient vibration suppression strategies has become a central challenge in the practical implementation of AMB technologies.

Existing vibration control strategies for magnetic bearings generally fall into two main categories: those that apply active suppression via unbalance compensation, and those that rely on dynamic self-balancing mechanisms. The unbalance compensation approach typically involves generating opposing magnetic forces through the bearings to neutralize the excitation caused by rotor mass asymmetry, thereby allowing the rotor to revolve more closely around its geometric centerline. These methods are often effective in scenarios requiring high precision and small air gaps. However, one notable limitation arises at higher rotor speeds: the resulting imbalance forces grow substantially, necessitating proportionally larger coil currents. This, in turn, can increase the burden on power amplifiers and raises the risk of current saturation, which compromises overall control performance.

Zhu’s research group made several noteworthy contributions to the development of unbalance compensation techniques for AMB rotors. In one early study [6] introduced an iterative method based on a quadrilateral search that estimates speed-independent unbalance parameters for determining compensatory electromagnetic forces. While promising, this method faced limitations in achieving both fast convergence and high accuracy. To address this, a subsequent improvement [7] incorporated a real-time triangular search with variable step sizes, which notably enhanced convergence speed. Later, a further refinement [8] proposed the use of variable search angles, aiming to mitigate the local-optimum entrapment often encountered with fixed-angle strategies. Although these advancements helped strike a better balance between precision and speed, they still struggled with computational efficiency—especially under conditions of variable rotor acceleration—and retained a relatively high level of algorithmic complexity.

In contrast to compensation-based methods, automatic balancing control focuses on suppressing unbalance-induced responses by shifting or filtering displacement signals detected by sensors. This effectively prevents the controller from reacting to such components, enabling the rotor to rotate around its inertia axis. Several strategies have been developed along this line. Tong et al. [9] introduced an optimal phase-compensated notch filter to improve stability across varying speeds. Liu et al. [10] extended this by designing a dual-input adaptive notch filter capable of suppressing synchronous currents over a wide operating range. Cui et al. [11], on the other hand, leveraged the orthogonality between *x*- and *y*-direction signals to simplify system integration. These notch-filter-based approaches collectively enhance vibration attenuation and system simplicity. Other researchers have pursued different paths. Yang et al. [12] applied a resonant controller with phase compensation to strengthen low-frequency stability. Bi et al. [13] transitioned from iterative learning to automatic learning control to achieve 0-displacement tracking via process switching, while Gao et al. [14] improved convergence speed. Filtering techniques also experienced development. Schuhmann et al. [15] integrated Kalman filters into the AMB framework, yielding notable performance gains. Repetitive control was explored by Han et al. [16], offering structural simplicity at the cost of requiring historical state storage. Beyond filtering and repetitive control, intelligent and adaptive techniques have drawn increasing attention. Tung et al. [17] proposed a fuzzy observer for unbalance force estimation and parameter adaptation. Ahmed et al. [18] employed adaptive feedback control to identify imbalance characteristics with encouraging results. Similarly, Zhang et al. [19] introduced an accelerated feedforward compensation strategy with model uncertainty correction, effectively reducing rotor vibrations under both harmonic and stochastic disturbances.

A notable limitation of many conventional control strategies lies in their tendency to overlook coupling effects between the multiple degree-of-freedom (DOF) in rotor systems. In some cases, inertial coupling is either underestimated or entirely ignored, which can lead to non-negligible control errors. Another common issue is their heavy reliance on precise mathematical models of the system. However, such models often fall short in capturing the real behavior of systems characterized by significant nonlinearities and time-varying dynamics. This discrepancy becomes especially pronounced in four-degree-of-freedom (4-DOF) magnetic bearing systems, where strong nonlinear interactions and parameter uncertainties are prevalent. To address these challenges, robust control techniques that can operate effectively under model uncertainties are essential. Among these, Active disturbance rejection control (ADRC) has gained substantial traction. It has demonstrated strong performance in handling nonlinear, time-varying, and multi-variable coupled systems, particularly in applications like motor control and rotor attitude stabilization [20]. The ADRC framework comprises three primary components: a tracking differentiator (TD), a nonlinear state error feedback (NLSEF) controller, and an extended state observer (ESO). Notably, ADRC treats both internal model deviations and external disturbances as a single “total disturbance,” which is estimated and compensated in real time using the ESO. This reduces dependence on accurate system modeling. Nevertheless, the ESO in its conventional form operates with a fixed bandwidth, which can limit its responsiveness to low-level or slowly varying disturbances, especially during transient phases [21].

In recent years, ADRC has attracted considerable interest in the field of AMB vibration suppression. This growing attention can be largely attributed to ADRC’s minimal reliance on precise system models and its ability to estimate and compensate for disturbances in real time. Despite its advantages, the traditional ESO used in ADRC frameworks still shows several limitations—particularly when dealing with high-frequency disturbances, tuning bandwidths, and managing coupled dynamics across multiple-DOF. For instance, the commonly used linear extended state observer (LESO) often suffers from phase lag and amplitude degradation at higher frequencies, which delays effective disturbance rejection [22]. Additionally, fixed-bandwidth settings fail to adapt to variations in rotor speed or changing operating environments, increasing the risk of current saturation or system instability. In response to these issues, researchers have proposed a range of enhancements. Gong et al. [23] introduced the enhanced extended state observer (ELESO), while Chai et al. [24] developed observer variants like adaptive-bandwidth and generalized extended state observer (GESO) that incorporate model information and enable real-time parameter adjustment or multiple-DOF decoupling. These developments have indeed improved vibration suppression to some extent. However, they still often depend on manually tuned parameters or simplified system assumptions. As a result, their effectiveness in handling strongly nonlinear and time-varying scenarios remains constrained [25,26].

Reinforcement learning techniques [27],—particularly the crested porcupine optimizer (CPO) algorithm—have recently emerged as promising tools for parameter tuning in complex control environments [28]. By incorporating explicit constraints during policy updates, CPO enables a control system to learn adaptive strategies through continuous interaction with the dynamic environment, while preserving system stability throughout the process [29,30]. These features make CPO especially well-suited for managing the nonlinearities, strong coupling, and fast-changing conditions inherent in AMB systems. Building on this insight, our work integrates the CPO framework into an enhanced linear active disturbance rejection control (ELADRC) scheme. This integration facilitates the adaptive tuning of critical controller parameters—such as observer bandwidth and feedback gains—helping to improve the trade-off between precise vibration suppression and energy efficiency. By doing so, we aim to overcome the limitations of conventional manual or trial-and-error tuning approaches.

This study presents a CPO-ELADRC strategy tailored for mitigating unbalance-induced vibration in AMB systems. By designing an adaptive observer to improve disturbance estimation and applying CPO for online controller adjustment, the proposed method demonstrates effective control performance across a wide range of rotor speeds. The approach not only enhances amplitude suppression and current stability but also offers a promising pathway toward high-efficiency and robust AMB operation under challenging conditions.

## 2. Modeling of Magnetic Bearing System

The chapter opens by constructing the dynamic model of the rotor system, where a linearized expression of the electromagnetic force is incorporated. This leads to the development of a complete set of system equations that capture the relationships among state variables, control inputs, and external disturbances. Such a formulation lays the groundwork for both the theoretical analysis and simulation-based validation of the proposed control strategy.

### 2.1. Rotor Dynamic Model

**Figure 1:**
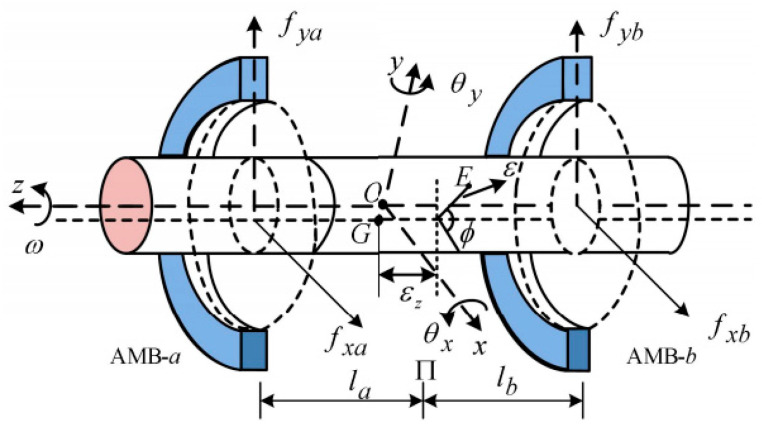
AMB Rotor System.

The rotor system of the AMB is illustrated in Figure 1. It primarily comprises a rotor, a pair of AMB positioned at both ends, and displacement sensors for feedback control. Point *O* represents the rotor’s mass center; $f_{xa}$, $f_{ya}$, $f_{xb}$, and $f_{yb}$ represent the electromagnetic forces acting in the *x*- and *y*-directions at the a and b ends, respectively; while *l_a_* and *l_b_* represents the distances from the geometric center *O* to the AMBs at ends a and b, respectively.

Based on rotor dynamics principles, the radial 4-DOF equations describing the lateral motion of AMB-supported rotors under the influence of mass unbalance can be formulated as follows:

(1) $$\left\{\begin{array}{c} J{\overset{••}{\theta }}_{y}-\overset{•}{\phi }J_{z}\overset{•}{\theta_{x}}=f_{xa}l_{a}-f_{xb}l_{b}+f_{1}\\ m\overset{••}{x}=f_{xa}+f_{xb}+f_{2}\\ J{\overset{••}{\theta }}_{x}+\overset{•}{\phi }J_{z}\overset{•}{\theta_{y}}=f_{yb}l_{b}-f_{ya}l_{a}+f_{3}\\ m\overset{••}{y}=f_{ya}+f_{yb}+f_{4} \end{array}\right.$$

where *x* and *y* represent the translational motions of the rotor’s geometric center *O*; *θ_x_* and *θ_y_* represent its angular displacements about the *x* and *y* axes, respectively; *m* represents the mass of the rotor; *J_x_*, *J_y_*, and *J_z_* represent the its moments of inertia along the *x*, *y* and *z* axes; while *f*_1_, *f*_2_, *f*_3_, and *f*_4_ represent the unbalance-induced excitations, which can be defined as follows:

(2) $$\left[\begin{array}{c} f_{1}\\ f_{2}\\ f_{3}\\ f_{4} \end{array}\right]=\left[\begin{array}{c} m_{e}\varepsilon ({\overset{•}{\phi }}^{2}\cos\phi +\overset{••}{\phi }\sin\phi )\varepsilon_{z}\\ m_{e}\varepsilon ({\overset{•}{\phi }}^{2}\cos\phi +\overset{••}{\phi }\sin\phi )\\ -m_{e}\varepsilon ({\overset{•}{\phi }}^{2}\sin\phi -\overset{••}{\phi }\cos\phi )\varepsilon_{z}\\ m_{e}\varepsilon ({\overset{•}{\phi }}^{2}\sin\phi -\overset{••}{\phi }\cos\phi ) \end{array}\right]$$

where *m*_e_ represents the unbalanced mass; *ε*, *ε*_z_, and ϕ represent the geometric characteristics of the unbalance; *ε* represents the radial offset; *ε*_z_ represents the axial offset; and ϕ represents the initial angular displacement of point *E* relative to the *x*-axis, measured from the rotor’s center of mass *O*.

Taking into account unbalance forces, inertial coupling, and gyroscopic effects, a dynamic model of the radial 4-DOF rotor system supported by magnetic bearings is developed. A coordinate system is defined with the rotor’s center of mass as the origin. Under this framework, the system’s equations of motion can be written as follows:

(3) $$M\overset{••}{q}+G\overset{•}{q}=L_{f}F+F_{d}$$

where $L_{f}=\left[\begin{array}{cccc} l_{a} & -l_{b} & 0 & 0\\ 1 & 1 & 0 & 0\\ 0 & 0 & -l_{a} & l_{b}\\ 0 & 0 & 1 & 1 \end{array}\right]$ represents the electromagnetic force allocation matrix; $G=\left[\begin{array}{cccc} 0 & 0 & -J_{z}\omega & 0\\ 0 & 0 & 0 & 0\\ J_{z}\omega & 0 & 0 & 0\\ 0 & 0 & 0 & 0 \end{array}\right]$ represents the gyroscopic matrix; $F=diag\left[f_{xa}f_{ya}f_{xb}f_{yb}\right]$ represents the electromagnetic force vector; $F_{d}={\left[f_{1}f_{2}f_{3}f_{4}\right]}^{T}$ represents the unbalance disturbance; $M=diag\left[\begin{array}{cccc} J & m & J & m \end{array}\right]$ represents the mass matrix; and $q={\left[x,y,\theta_{x},\theta_{y}\right]}^{T}$ represents the vector of displacements and rotational angles.

Taking the rotor’s motion along the *x*-axis at end *a* as an example, the system is affected not only by the local electromagnetic force in the *x*-direction at end *a*, but also by the coupled effects from the electromagnetic force at end *b*, lateral coupling in the y-direction, and the unbalance excitation.

### 2.2. Electromagnetic Force Linearization

Building upon the 4-DOF rotor dynamics model, the electromagnetic force functions as the primary actuation input in AMB control. Its dependence on both control current and air-gap displacement plays a pivotal role in determining the system’s control precision and overall stability. Notably, the magnetic force exhibits strong nonlinearity, being inversely proportional to the square of the air gap and directly proportional to the square of the current. However, during stable rotor suspension, displacement deviations remain relatively small. To facilitate controller design and enhance real-time disturbance compensation, the nonlinear electromagnetic force expression is linearized around the operating point. This approximation yields a linear time-invariant representation, forming the basis for subsequent LESO-based disturbance estimation and CPO-driven parameter adaptation.

Sensor misalignment is not considered in this study. All AMB operate under a differential drive scheme, in which opposing magnetic poles along each axis are energized using a fixed bias current and a variable control current applied in opposite directions. Given the small rotor displacement during stable levitation, the electromagnetic force can be linearized around the equilibrium position. Considering the *x*-axis at one rotor end as an example, the electromagnetic force *f_ax_* can be approximated as follows:

(4) $$f_{ax}=k_{i}i_{xa}+k_{s}x_{xa}$$

where *k_i_* represents the current stiffness coefficient; *k_s_* represents the displacement stiffness coefficient; *i* represents the control current; and *x* represents the displacement deviation. Combining Equations (1)–(4), the following relationship can be obtained:

(5) $$\left\{\begin{array}{c} {\overset{••}{x}}_{a}=a_{0}x_{a}+a_{1}i_{xa}+d_{xa}\\ {\overset{••}{x}}_{b}=a_{0}x_{b}+a_{1}i_{xb}+d_{xb}\\ {\overset{••}{y}}_{a}=a_{0}y_{a}+a_{1}i_{ya}+d_{ya}\\ {\overset{••}{y}}_{b}=a_{0}y_{b}+a_{1}i_{yb}+d_{yb} \end{array}\right.$$

where $a_{0}$ represents the combined coefficient of displacement stiffness and geometric parameters; $a_{1}$ represents the combined coefficient of current stiffness and geometric parameters; while *d_xa_*, *d_xb_*, *d_ya_*, and *d_yb_* represent the lumped disturbance acting on the 4-DOF, in addition to the rotor’s controllable electromagnetic force.

The preceding modeling process integrates mechanical kinematics with electromagnetic coupling to establish a comprehensive mathematical description of the rotor system. A 4-DOF dynamic formulation was used to capture the effects of unbalanced mass excitation, gyroscopic moments, and inertial coupling on system vibrations. Through Taylor expansion around the equilibrium point, the inherently nonlinear electromagnetic force expression was linearized, resulting in a formulation defined by the *k_i_* and *k_s_*. Traditional vibration suppression strategies such as PID control suffer from strong model dependency and limited adaptability. Their reliance on empirical tuning makes them inadequate for handling multiple-DOF coupling and the nonlinear characteristics of magnetic actuation, especially across wide speed ranges. While the LADRC framework improves disturbance estimation via the LESO, its fixed-bandwidth configuration struggles to accommodate frequency shifts caused by speed variations—leading to delayed compensation of unbalanced forces. To address these limitations, this study introduces a CPO-ELADRC strategy. Drawing inspiration from the multimodal defensive behavior modeled in the CPO, this approach enables adaptive tuning of both the $\omega_{0}$ and $\omega_{c}$, thereby enhancing responsiveness under varying operational conditions. It is worth noting that, due to the linear relationship between control current and electromagnetic force, the unbalance force disturbance described in Equation (2) can be equivalently represented as a sinusoidal current disturbance acting through the electromagnetic force channel. This equivalence provides a convenient framework for disturbance.

## 3. Design of a CPO-ELADRC Strategy for Unbalance Vibration Suppression in Magnetic Bearings

The established 4-DOF magnetic bearing rotor model exhibits significant nonlinear electromagnetic coupling, strong multi-axis dynamic interactions, and sensitivity to time-varying disturbances across a wide speed range. These characteristics place high demands on the controller’s robustness and adaptability. Conventional PID control methods, which rely heavily on accurate system modeling, face difficulties in decoupling multivariable dynamics. While standard LADRC offers improved disturbance rejection through a LESO, its fixed-bandwidth design cannot adapt to frequency shifts induced by speed changes—leading to lagging compensation and diminished high-frequency response.

This chapter introduces a composite control strategy—CPO-ELADRC. The proposed framework integrates intelligent optimization and advanced disturbance rejection to improve vibration suppression performance under time-varying and nonlinear conditions. Firstly, the LESO performs real-time estimation of total system disturbances, enabling feedforward compensation to attenuate unmodeled dynamics. Secondly, the CPO, inspired by multimodal search mechanisms, dynamically tunes the LESO bandwidth and LADRC control gains. This adaptive adjustment helps maintain a balance between vibration attenuation precision and control effort across a wide speed range. Thirdly, system states—including rotor displacement, rotational speed, and control current—are integrated into a reward function that quantitatively reflects the trade-off between performance and energy consumption. Finally, based on reward-driven feedback, the equivalent stiffness and damping matrices of the control system are modulated in real time to enhance robustness and adaptability under varying operating conditions.

### 3.1. LADRC

**Figure 2:**
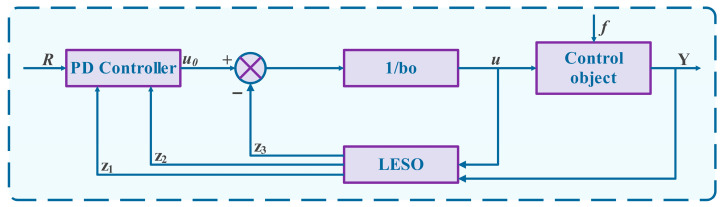
LADRC Basic structure diagram.

LADRC is valued for its low dependency on accurate system models. Instead of relying on precise mathematical descriptions, it employs a LESO to estimate total disturbances—including external perturbations and internal uncertainties—in real time. These disturbances are then actively compensated through feedforward mechanisms, effectively converting the system into a controllable series-integral form. Compared to the original ADRC formulation, LADRC introduces the concept of bandwidth as a key design parameter. This not only simplifies controller tuning by reducing the number of adjustable parameters but also enhances implementation efficiency. The overall structure of LADRC is illustrated in Figure 2.

The resulting LADRC-based augmented model is given by:

(6) $$\left\{\begin{array}{c} \overset{••}{y}=-\alpha \overset{•}{y}+\beta y+bu+d\\ x_{1}=y\\ x_{2}=\dot{y} \end{array}\right.$$

where *α* and *β* represent the known system structure parameters, determined by the electromagnetic stiffness and damping coefficients; *b* represents the control input gain, which is related to the *k_i_*; u represents the control current; and *f* represents the total disturbance.

The corresponding extended state-space formulation is described as follows:

(7) $$\begin{array}{l} \left[\begin{array}{c} \overset{•}{x_{1}}\\ \overset{•}{x_{2}} \end{array}\right]=\left[\begin{array}{cc} 0 & 1\\ \beta & -\alpha \end{array}\right]\left[\begin{array}{c} x_{1}\\ x_{2} \end{array}\right]+\left[\begin{array}{c} 0\\ b \end{array}\right]u+\left[\begin{array}{c} 0\\ 1 \end{array}\right]d\\ y=\left[\begin{array}{cc} 1 & 0 \end{array}\right]\left[\begin{array}{c} x_{1}\\ x_{2} \end{array}\right] \end{array}$$

The controller is formulated as follows:

(8) $$u=-\left[\begin{array}{cc} K_{1} & K_{2} \end{array}\right]\left[\begin{array}{c} x_{1}\\ x_{2} \end{array}\right]+F_{r}$$

where *F_r_* represents the feedforward term; while *K*_1_ and *K*_2_ represent the gain coefficients of the state error feedback control law. When *x*_1_ and *x*_2_ are not directly measurable, a state observer is employed to estimate their values. Consequently, Equation (9) can be expressed as follows:

(9) $$u=-\left[\begin{array}{cc} K_{1} & K_{2} \end{array}\right]\left[\begin{array}{c} \hat{x_{1}}\\ \hat{x_{2}} \end{array}\right]+F_{r}$$

where $\hat{x_{1}}$ and $\hat{x_{2}}$ represent the estimated states provided by the observer.

The introduced closed-loop observer (i.e., the Luenberger observer) is represented by the following dynamic structure:

(10) $$\overset{•}{\hat{x}}=A\hat{x}+Bu+\left[\begin{array}{c} L_{1}\\ L_{2} \end{array}\right](y-\hat{y})$$

where A represents the system matrix; $\hat{x}$ represents the estimated state vector; *B* represents the input matrix; and $\left[\begin{array}{c} L_{1}\\ L_{2} \end{array}\right]$ represents the observer gain matrix.

#### 3.1.1. Design of a Model-Free LESO

The dynamic model of the LESO, used for estimating system states and disturbances, is formulated as follows:

(11) $$\left\{\begin{array}{c} \overset{••}{y}=-\alpha \overset{•}{y}+\beta y+b_{0}u+d\\ x_{1}=y\\ x_{2}=\overset{•}{y}\\ x_{3}=f \end{array}\right.$$

where *x*_3_ represents the extended state and *f* represents the total disturbance, which comprises both external influences and internal uncertainties—such as unmodeled dynamics or parameter deviations. By aggregating all non-output dynamics into this disturbance term, Equation (11) is expressed as follows:

(12) $$f=-\alpha \overset{•}{y}+\beta y+d$$

Accordingly, the system dynamics can be reformulated in an augmented state-space form, where the total disturbance is modeled as an extended state, as follows:

(13) $$\begin{array}{l} \left[\begin{array}{c} \overset{•}{x_{1}}\\ \overset{•}{x_{2}}\\ \overset{•}{x_{3}} \end{array}\right]=\left[\begin{array}{ccc} 0 & 1 & 0\\ 0 & 0 & 1\\ 0 & 0 & 0 \end{array}\right]\left[\begin{array}{c} x_{1}\\ x_{2}\\ x_{3} \end{array}\right]+\left[\begin{array}{c} 0\\ b_{o}\\ 0 \end{array}\right]u\\ y=\left[\begin{array}{ccc} 1 & 0 & 0 \end{array}\right]\left[\begin{array}{c} x_{1}\\ x_{2}\\ x_{3} \end{array}\right] \end{array}$$

Based on Equation (10), the observer structure can be derived as follows to support state and disturbance estimation:

(14) $$\left\{\begin{array}{c} \overset{•}{\hat{x}}=A\hat{x}+Bu+L(y-\hat{y})\\ \hat{y}=C\hat{x} \end{array}\right.$$

where $A=\left[\begin{array}{ccc} 0 & 1 & 0\\ 0 & 0 & 1\\ 0 & 0 & 0 \end{array}\right],\;B=\left[\begin{array}{c} 0\\ b_{o}\\ 0 \end{array}\right],\;C=\left[\begin{array}{ccc} 1 & 0 & 0 \end{array}\right],\;L=\left[\begin{array}{c} l_{1}\\ l_{2}\\ l_{3} \end{array}\right]$, and C represents the output matrix.

From the state-space relation, the following expression can be derived for clarity:

(15) $$\overset{•}{\hat{x}}=\left[\begin{array}{ccc} l_{1} & 1 & 0\\ l_{2} & 0 & 1\\ l_{3} & 0 & 0 \end{array}\right]\hat{x}+\left[\begin{array}{cc} 0 & l_{1}\\ b_{o} & l_{2}\\ 0 & l_{3} \end{array}\right]\left[\begin{array}{c} u\\ y \end{array}\right]$$

By computing the eigenvalues of the system matrix $\overset{—}{A}=\left[\begin{array}{ccc} l_{1} & 1 & 0\\ l_{2} & 0 & 1\\ l_{3} & 0 & 0 \end{array}\right]$, the characteristic polynomial can be expressed in terms of the *l*_1_, *l*_2_, *l*_3_ as follows:

(16) $$SI-\overset{—}{A}=\left[\begin{array}{ccc} s & 0 & 0\\ 0 & s & 0\\ 0 & 0 & s \end{array}\right]-\left[\begin{array}{ccc} l_{1} & 1 & 0\\ l_{2} & 0 & 1\\ l_{3} & 0 & 0 \end{array}\right]=s^{3}+l_{1}s^{2}+l_{2}s+l_{3}$$

**Figure 3:**
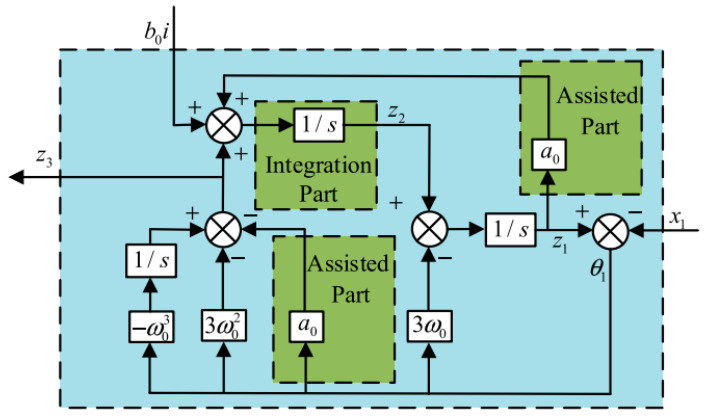
LESO Structure.

The roots of the characteristic polynomial determine the observer’s dynamic response speed and stability. To ensure all roots lie in the left-half of the complex plane, pole placement is employed. For tuning convenience, the observer poles are placed at real locations denoted by $-\omega_{0}$, where $\omega_{0}$ represents the LESO bandwidth. Accordingly, based on the above derivation, the gain parameters *l*_1_, *l*_2_, *l*_3_ can be expressed as functions of $\omega_{0}$:

(17) $$s^{3}+l_{1}s^{2}+l_{2}s+l_{3}={\left[S-(-\omega_{o})\right]}^{3}=s^{3}+3\omega_{o}s^{2}+3\omega_{o}^{2}s+\omega_{o}^{3}$$

where $l_{1}=3\omega_{o},\;l_{2}=3\omega_{o}^{2},\;l_{3}=\omega_{o}^{3}$. In the LESO design, the three observer gain parameters are unified into a $\omega_{0}$. To enhance adaptability, the $\omega_{0}$ is dynamically optimized via the CPO algorithm, enabling automatic balance between disturbance rejection performance and control effort. Consequently, the observer can be tuned by adjusting only the $\omega_{0}$, which governs the overall system response speed. The established LESO structure is illustrated in Figure 3.

#### 3.1.2. Design of the State Error Feedback Control Law

When the designed state observer can accurately estimate the total disturbance, i.e., when $\hat{x_{3}}=\hat{f}\approx f$ = $u=\frac{u_{0}-\hat{f}}{b_{0}}$. Then Equation (18) can be determined as follows:

(18) $$\overset{••}{y}=f+bu=f+b\frac{u_{0}-\hat{f}}{b_{0}}=u_{0}$$

where *b*_0_ represents the estimated value of the control gain *b*, It is computed based on the pre-control input terms derived from the established system model, with the goal of closely approximating the true value of *b*. Any deviation between *b*_0_ and the actual *b* is absorbed into the total disturbance term *f* and subsequently estimated by the observer. Therefore, improving the accuracy of *b*_0_ effectively reduces the disturbance estimation burden on the LESO.

As a result, the equation can be reformulated as follows:

(19) $$\overset{••}{y}=-\alpha \dot{y}+\beta y+\Delta u+d+b_{o}u$$

where $f=-\alpha \dot{y}+\beta y+\Delta u+d,\Delta u=bu-b_{o}u$.

After incorporating all parameters unrelated to the control input *u* into the total disturbance *f*, the LESO is capable of fully estimating and compensating for these effects. As a result, the system can be reformulated into a series-integral canonical form, enabling the original PD controller to achieve precise regulation.

Accordingly, Equation (18) can be rewritten as:

(20) $$u_{o}=K_{p}(r-\hat{x_{1}})+K_{d}(\overset{•}{r}-\hat{x_{2}})+\overset{••}{r}$$

where *r* represents the reference input and $\overset{••}{r}$ represents a feedforward gain to improve the system’s reference tracking performance.

The above equation can be transformed into the Laplace domain as follows:

(21) $$s^{2}Y=K_{p}\cdot E+K_{d}\cdot s\cdot E$$

From the above derivation, the open-loop transfer function $\frac{Y}{E}=\frac{K_{p}+K_{d}\cdot s}{s^{2}}$ is obtained. Accordingly, the corresponding closed-loop transfer function is given by:

(22) $$G(s)=\frac{\frac{Y}{E}}{1+\frac{Y}{E}}=\frac{K_{p}+K_{d}\cdot s}{s^{2}+K_{d}\cdot s+K_{p}}$$

The controller is designed via pole placement, with $-\omega_{c}$ selected as the bandwidth parameter to uniformly shape the dynamic response of the closed-loop system. Based on this configuration, the following can be derived:

(23) $$s^{2}+K_{d}\cdot s+K_{p}={\left[s-(-\omega_{o})\right]}^{2}=s^{2}+2\omega_{c}s+\omega_{c}^{2}$$

$∴K_{p}=\omega_{c}^{2},K_{d}=2\omega_{c}$. In this way, the entire controller can be tuned by adjusting only the bandwidth parameter $\omega_{c}$. By dynamically optimizing $\omega_{c}$ using the CPO algorithm, the system achieves adaptive parameter coordination across a wide range of rotor speeds.

### 3.2. Design of LESO for AMB

**Figure 4:**
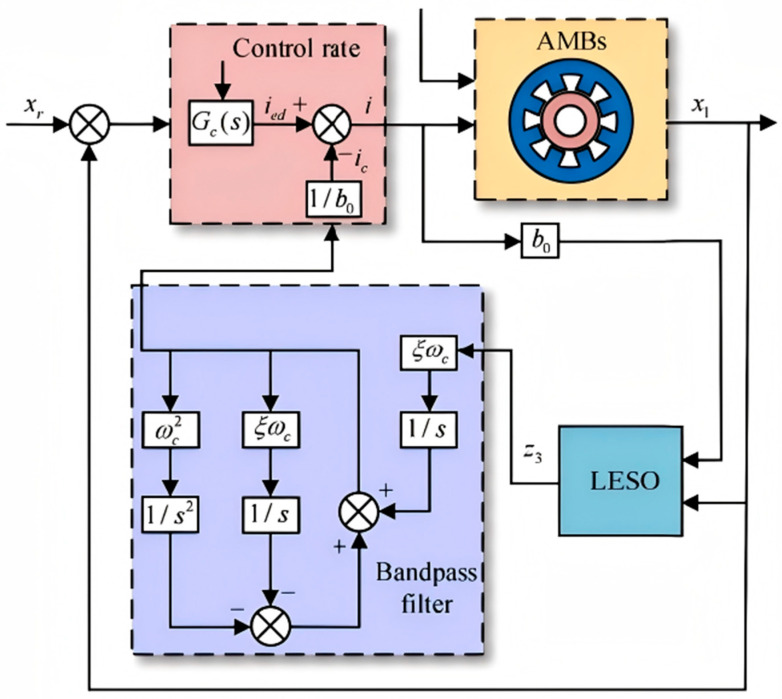
Control diagram of single-DOF AMB rotor system.

Substituting the AMB model (Figure 4) into Equation (18), the system can be formulated as follows:

(24) $$\overset{••}{y}=-a_{1}\dot{y}-a_{0}y+\Delta u+d+b_{o}u=-a_{1}\dot{y}-a_{0}y+f^{\prime }+b_{o}u$$

where *a*_0_ and *a*_1_ are known, *b*_0_ is partially known, and $f^{\prime }=\Delta u+d$ represents the actually unknown disturbance, which is still considered as the total disturbance.

Accordingly, the state-space equation incorporating the extended state can be expressed as:

(25) $$\left\{\begin{array}{c} \dot{x}=Ax+Bu+E{\dot{f}}^{\prime }\\ y=Cx \end{array}\right.\;\;f=a_{1}\dot{y}+a_{0}y+\Delta u+d$$

Accordingly, the system matrices in the extended state-space representation can be defined as follows:

(26) $$A={\left[\begin{array}{ccc} 0 & 1 & 0\\ 0 & 0 & 1\\ 0 & -a_{0} & -a_{1} \end{array}\right]}_{,}B={\left[\begin{array}{c} 0\\ b_{o}\\ -a_{1}b_{o} \end{array}\right]}_{,}C={\left[\begin{array}{ccc} 1 & 0 & 0 \end{array}\right]}_{,}E=\left[\begin{array}{c} 0\\ 0\\ 1 \end{array}\right]$$

Therefore, the observer dynamics can be formulated as follows:

(27) $$\left\{\begin{array}{c} \overset{•}{\hat{x}}=(A-LC)\hat{x}+Bu+Ly+E{\overset{•}{f}}^{\prime }\\ \hat{y}=C\hat{x} \end{array}\right.$$

Following the same eigenvalue calculation method used in Equation (17), we obtain,

(28) $$\begin{array}{l} SI-\overset{—}{A}=\left[\begin{array}{ccc} S & 0 & 0\\ 0 & S & 0\\ 0 & 0 & S \end{array}\right]-\left[\begin{array}{ccc} l_{1} & 1 & 0\\ l_{2} & 0 & 1\\ l_{3} & -a_{0} & -a_{1} \end{array}\right]\\ =S^{3}+l_{1}S^{2}-a_{1}S^{2}-a_{1}l_{1}S-a_{0}S+l_{2}S-a_{0}l_{1}-a_{1}l_{2}+l_{3} \end{array}$$

The observer poles are placed in the left half of the complex plane, and for ease of tuning, all poles are set as $-\omega_{0}$.

(29) $$\begin{array}{l} S^{3}+l_{1}S^{2}-a_{1}S^{2}-a_{1}l_{1}S-a_{0}S+l_{2}S-a_{0}l_{1}-a_{1}l_{2}+l_{3}\\ ={\left[S-(-\omega_{o})\right]}^{3}\\ =S^{3}+(3\omega_{o}-a_{1})S^{2}+(3\omega_{o}^{2}-a_{1}\omega_{o}-a_{0}+a_{1}^{2})S+\omega_{o}^{3}-3a_{1}\omega_{o}^{2}+3(a_{1}^{2}-a_{0})\omega_{o}+2a_{0}a_{1}-a_{1}^{3} \end{array}$$

This yields the following result:

(30) $$\begin{array}{l} l_{1}=3\omega_{o}-a_{1}\\ l_{2}=3\omega_{o}^{2}-a_{1}\omega_{o}-a_{0}+a_{1}^{2}\\ l_{3}=\omega_{o}^{3}-3a_{1}\omega_{o}^{2}+3(a_{1}^{2}-a_{0})\omega_{o}+2a_{0}a_{1}-a_{1}^{3} \end{array}$$

In Equation (30), all parameters except the $\omega_{0}$ are known from the system model. Therefore, the observer can be fully parameterized using the $\omega_{0}$, which significantly simplifies tuning. This formulation serves as the foundation for the core control framework of the proposed CPO-ELADRC strategy.

### 3.3. CPO Parameter Optimization

**Figure 5:**
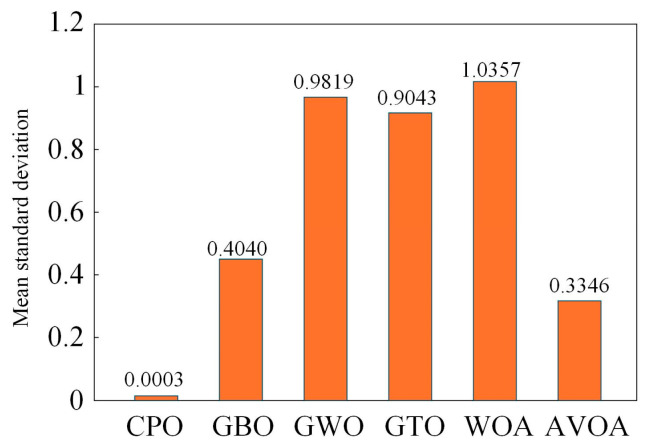
Average Standard Deviation of CEC2017.

CPO belongs to the class of bio-inspired metaheuristic algorithms derived from natural phenomena, and its design principle is motivated by the adaptive defense mechanisms exhibited by crested porcupine populations in complex ecological environments. Using the CEC2017 benchmark suite as a test case, Mohamed Abdel-Basset evaluated the performance of CPO in comparison with several classical metaheuristic algorithms. The resulting mean and standard deviation values, as illustrated in Figure 5, demonstrate the superior performance of the CPO algorithm.

**Figure 6:**
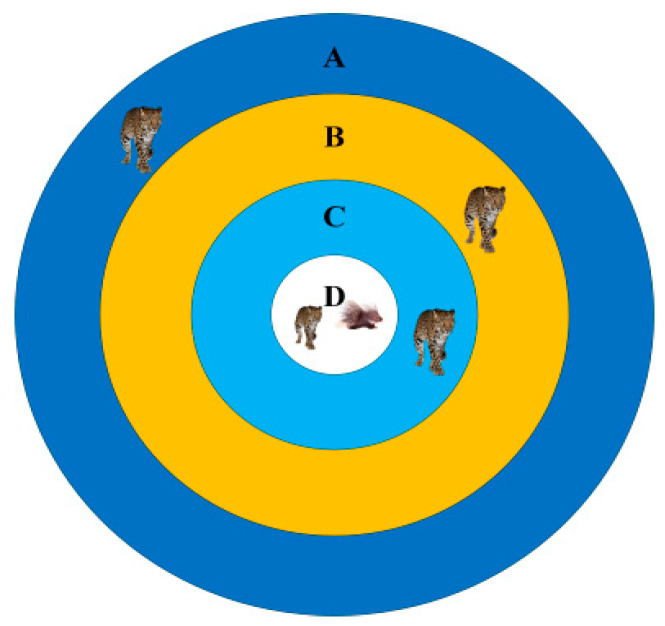
Defensive Range of Crested Porcupine (Hystrix cristata).

The proposed CPO-based multi-modal defense mechanism is inspired by the four-stage threat response behavior of the crested porcupine, as illustrated in Figure 6. The hierarchical defense search space is divided into four regions (A–D) according to a decaying threat radius, representing increasing response intensity. Region A corresponds to global exploration, while Regions B and C progressively enhance local search and global diversification through adaptive interaction mechanisms. Region D represents the final intensive exploitation phase. Together, these regions guide the CPO algorithm to achieve a balanced transition between exploration and exploitation in complex nonlinear optimization problems.

The core innovation of the CPO algorithm lies in its cyclical population reduction mechanism, which effectively preserves population diversity while enhancing convergence speed. This mechanism adaptively adjusts the population size over iterations, striking a dynamic balance between global exploration and local exploitation. The corresponding mathematical model is formulated as follows:

(31) $$N=N_{\min}+(N^{\prime }-N_{\min})\times \left(1-\left(\frac{t\%\frac{T_{\max}}{T}}{\frac{T_{\max}}{T}}\right)\right)$$

where *N* represents the population size; *T* represents the variable controlling the cyclical iterations; *T*_max_ represents the maximum number of function evaluation iterations; *N*_min_ represents the minimum number of individuals in the newly generated population; and *t* represents the current iteration number.

Compared to other animals, the crested porcupine is characterized by a uniquely specialized predator-defense strategy. The CPO algorithm is explicitly inspired by this multi-stage defensive behavior and is executed in two primary phases: global exploration and local exploitation.

(1) Global Exploration Phase: The crested porcupine maintains a relatively large distance from the predator and primarily relies on visual and acoustic deterrent strategies.

As the first line of defense, the porcupine raises and fans its quills to warn the predator, simulating a visual threat. This behavior corresponds to the following mathematical model:

(32) $$x_{i}^{t+1}=x_{i}^{t}+\tau_{1}\times \left|2\times \tau_{2}\right.\times x_{CP}^{t}-\left.y_{i}^{t}\right|$$

As the second line of defense, the crested porcupine further deters the approaching predator by generating threatening noises. This acoustic warning behavior can be represented by the following mathematical model:

(33) $$x_{i}^{t+1}=(1-U_{1})\times x_{i}^{t}+U_{1}\times \left(y+\tau_{3}\times (x_{r1}^{t}-x_{r2}^{t})\right)$$

where $x_{i}^{t}$ represents the position of the *i*-th individual at iteration *t*; $x_{i}^{t+1}$ represents the position of the *i*-th individual at the next iteration; $\tau_{1}$ represents a normally distributed random variable; $\tau_{2}$ and $\tau_{3}$ represent the random numbers in the range [0, 1]; $x_{CP}^{t}$ represents the current global best solution of the objective function; $y_{i}^{t}$ represents the position of the predator at iteration *t*; *U*_1_ represents a binary vector composed of 0 s and 1 s; while *r*1 and *r*2 represent the two random integers in the range [1, N].

(2) Local Exploitation Phase: When the predator is in close proximity, the porcupine adopts more aggressive defensive strategies, including olfactory diffusion and physical attack.

As the third line of defense, the porcupine emits a strong odor that disperses throughout the surrounding environment, creating a chemical deterrent effect. This behavior can be described by the following mathematical model:

(34) $$x_{i}^{t+1}=(1-U_{1})\times x_{i}^{t}+U_{1}\times (x_{r1}^{t}+S_{i}^{t}\times (x_{r2}^{t}-x_{r3}^{t})-\tau_{3}\times \delta \times \gamma_{t}\times S_{i}^{t})$$

As the fourth and final line of defense, if the preceding mechanisms fail to deter the predator, the porcupine resorts to a direct physical attack. This aggressive behavior is modeled to simulate a decisive local exploitation response within the CPO algorithm. The corresponding mathematical model is given by:

(35) $$x_{i}^{t+1}=x_{CP}^{t}+(\alpha (1-\tau_{4})+\tau_{4})\times (\delta \times x_{CP}^{t}-x_{i}^{t})-\tau_{5}\times \delta \times \gamma_{1}\times F_{i}^{t}$$

where *r*3 represents a random integer in the range [1, N]; $\gamma_{t}$ represents the defense factor; $S_{i}^{t}$ represents the odor diffusion factor; δ represents the parameter controlling the search direction; α represents the convergence rate factor; $\tau_{3}$ and $\tau_{4}$ represent the random numbers in the range [0, 1]; and $F_{i}^{t}$ represents the inelastic collision force generated when an individual physically attacks the predator.

Based on the underlying principles of the CPO algorithm, the global exploration–local exploitation behavior of the porcupine can be used to guide the $\omega_{0}$ and $\omega_{c}$. By simulating this multi-modal defensive process, a four-stage optimization framework is constructed, enabling adaptive adjustment of these parameters across different search phases. The detailed optimization procedure is as follows:

(1) Population Initialization: Randomly generate 20 candidate solutions for the parameter ${\left\{\omega_{0}^{k},\omega_{c}^{k}\right\}}_{k-1}^{20}$, ensuring coverage of its entire feasible domain.

(2) Global Exploration Phase: Simulate the porcupine’s quill-fanning behavior to expand the perceived threat range. This is achieved by perturbing the initial population to explore diverse regions of the search space, thereby generating new candidate solutions with enhanced global diversity.

(36) $$\omega_{0}^{k}=\omega_{0}^{\min}+\mathrm{rand}(1)\cdot (\omega_{0}^{\max}-\omega_{0}^{\min})$$

where rand(1) represents a uniformly distributed random number, ensuring coverage of the global search space. In the second defense stage, a neighborhood information exchange mechanism is introduced to enhance the diversity. The resulting position update rule is defined as follows:

(37) $$\omega_{c}^{k}=\omega_{c}^{k}+\gamma \cdot (\omega_{c}^{best}-\omega_{c}^{k})+\delta \cdot N(0,1)$$

where γ = 0.3 represents the social learning factor; δ = 15 represents the disturbance intensity; and $\omega_{c}^{best}$ represents the current optimal solution.

(3) Local Exploitation Phase: The search range is gradually contracted around the current optimal solution $\left\{\omega_{0}^{*},\;\omega_{c}^{*}\right\}$. This contraction is regulated by the defense factor $\eta (t)=1-\frac{t}{T_{\max}}$ (where *t* = the current iteration and *T*_max_ = 500) as follows:

(38) $$\omega_{0}^{k}=\omega_{0}^{*}+\eta (t)\cdot \varepsilon \cdot (\omega_{0}^{r}-\omega_{0}^{k})$$

where ε = 0.2 represents the diffusion factor and $\omega_{0}^{k}$ represents a randomly selected individual from the population, simulating aggregation behavior driven by odor concentration gradients.

(4) Physical Attack Phase: Candidate solutions with low fitness are subjected to forced perturbations. This mechanism mimics a direct physical strike and aims to rapidly escape local optima. The position update rule, guided by the gradient of the objective function, is defined as follows:

(39) $$\omega_{c}^{k}=\omega_{c}^{k}-\zeta \cdot \nabla R(\omega_{c}^{k})$$

where ζ = 0.01 represents the learning rate and ∇R is calculated using the finite difference method as follows: $\nabla R\approx [R(\omega_{c}^{k}+\Delta )-R(\omega_{c}^{k})]/\Delta (\Delta =10)$.

(5) Convergence Criterion: The optimization process is considered to have converged when the variance $Var(R)\;<\;10^{-4}$ of the reward function over 50 consecutive iterations falls below a predefined threshold. At this point, the corresponding optimal parameter $\left\{\omega_{0}^{*},\;\omega_{c}^{*}\right\}$ is selected as the final output.

### 3.4. Design of CPO-ELADRC Strategy

Based on the principles of the CPO and taking into account the strong coupling and time-varying disturbance characteristics inherent in magnetic bearing rotor systems, this section proposes a collaborative optimization framework that integrates CPO with the ELADRC strategy. The proposed framework enables multi-objective optimization by dynamically tuning key control parameters to strike a balance between vibration suppression performance and control energy efficiency. Regarding the optimization process, the sensitivity of controller performance to key parameters is primarily reflected in the observer bandwidth and feedback gains, which directly affect disturbance estimation accuracy and dynamic response. The CPO algorithm exhibits stable convergence behavior under the selected population size and iteration number, as the optimization dimension is limited and the search space is well constrained. Moreover, the computational burden of the CPO is relatively moderate in this application, since the optimization is performed with a small number of parameters and iterations. Therefore, the proposed optimization strategy achieves a reasonable balance between performance improvement and computational efficiency. Guided by the structural characteristics of the ELADRC controller and the dynamic behavior of the magnetic bearing system, the core parameters subject to optimization are summarized in Table 1.

#### 3.4.1. CPO-ELADRC Algorithm Framework

**Figure 7:**
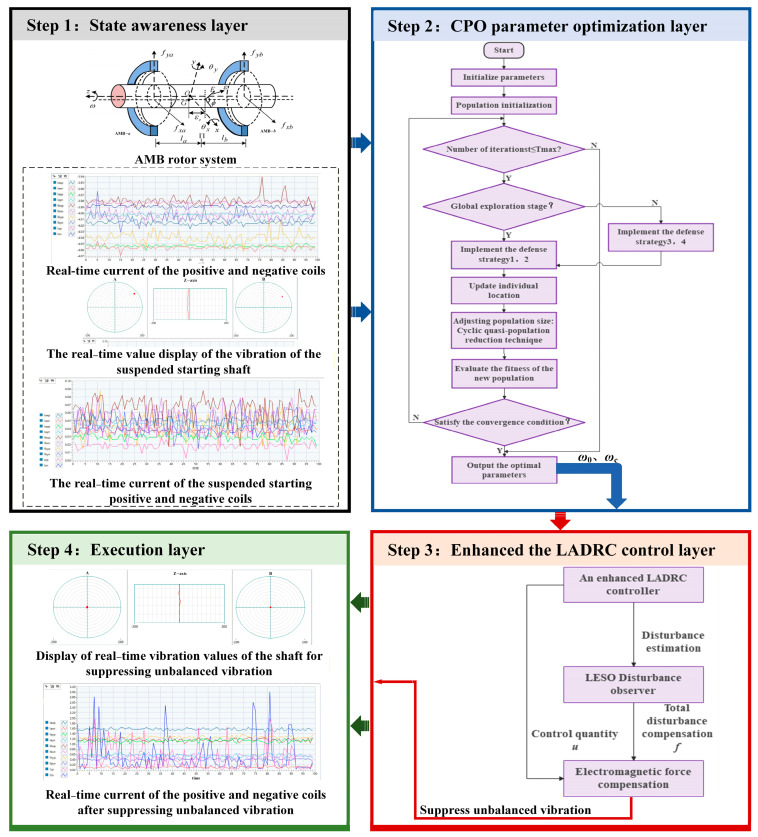
CPO-ELADRC Algorithm Framework.

The overall framework of the proposed CPO-ELADRC algorithm is illustrated in Figure 7. By integrating the CPO with ELADRC, the system achieves effective vibration suppression for magnetic bearing rotors through dynamic parameter optimization and real-time disturbance compensation. The architecture consists of four hierarchical layers: the state perception layer, which acquires system variables such as rotor displacement, rotational speed, and control current; the parameter optimization layer, where the CPO algorithm dynamically adjusts key LADRC parameters based on the reward function; the control decision-making layer, which computes control inputs based on optimized LESO and LADRC outputs; and the execution layer, where compensatory control signals are applied to the actuator in real time. In this framework, CPO addresses the limitation of conventional LADRC with fixed controller parameters by continuously tuning core parameters (e.g., $\omega_{0}$ and $\omega_{c}$) based on system dynamics. The LESO, equipped with optimized $\omega_{0}$, enhances the accuracy of total disturbance estimation. This results in improved feedforward compensation, forming a dual closed-loop structure that tightly integrates disturbance observation, compensation, and feedback regulation. The reward function guiding CPO optimization incorporates two competing objectives: vibration suppression, achieved through displacement minimization, and energy efficiency, realized by minimizing control current. By balancing these goals, the framework effectively prevents control saturation and excessive energy consumption, thus maintaining high control performance across varying operational conditions.

The proposed CPO-ELADRC composite control strategy achieves vibration suppression in magnetic bearing systems through a hierarchical collaborative mechanism. The state sensing layer continuously acquires critical rotor states—including displacement, rotational speed, and control current—and constructs a multiple-DOF state-space vector to provide real-time input for downstream control decisions. The CPO-based parameter optimization layer, inspired by the crested porcupine’s multi-modal defense behavior, dynamically adjusts the $\omega_{0}$ and $\omega_{c}$ via global exploration and local exploitation, thereby achieving a trade-off between vibration suppression precision and control energy consumption. The ELADRC layer utilizes these optimized parameters to configure a generalized LESO, which estimates total disturbances in real time and compensates them through feedforward control. Simultaneously, feedback control modulates the system’s stiffness and damping matrices, enhancing the robustness and responsiveness of the closed-loop system under time-varying operating conditions. The execution layer converts control commands into drive currents via an electromagnetic force compensation module, generating electromagnetic forces that are equal in magnitude and opposite in phase to the estimated disturbances, thereby actively suppressing rotor vibrations. Through coordinated state feedback and adaptive parameter adjustment across all layers, the strategy forms a tightly coupled closed-loop system that ensures robust disturbance rejection and stable operation across a wide range of rotational speeds.

Conventional PID control relies on fixed-parameter feedback and lacks explicit disturbance modeling, which limits its robustness under varying operating conditions. LADRC improves robustness by introducing an extended state observer to estimate lumped disturbances; however, its performance strongly depends on manual parameter tuning and fixed observer bandwidth selection. ELADRC further enhances adaptability by incorporating nonlinear error feedback, yet its tuning process remains experience-dependent and may not be optimal across wide operating ranges. In contrast, the proposed CPO-ELADRC framework integrates metaheuristic optimization with ELADRC parameter tuning, enabling adaptive and systematic adjustment of controller and observer parameters according to system dynamics. This mechanism allows the controller to better balance disturbance rejection, noise sensitivity, and dynamic response, thereby providing a clear theoretical basis for the observed performance improvements beyond purely numerical gains.

#### 3.4.2. The CPO-ELADRC Algorithm

**Figure 8:**
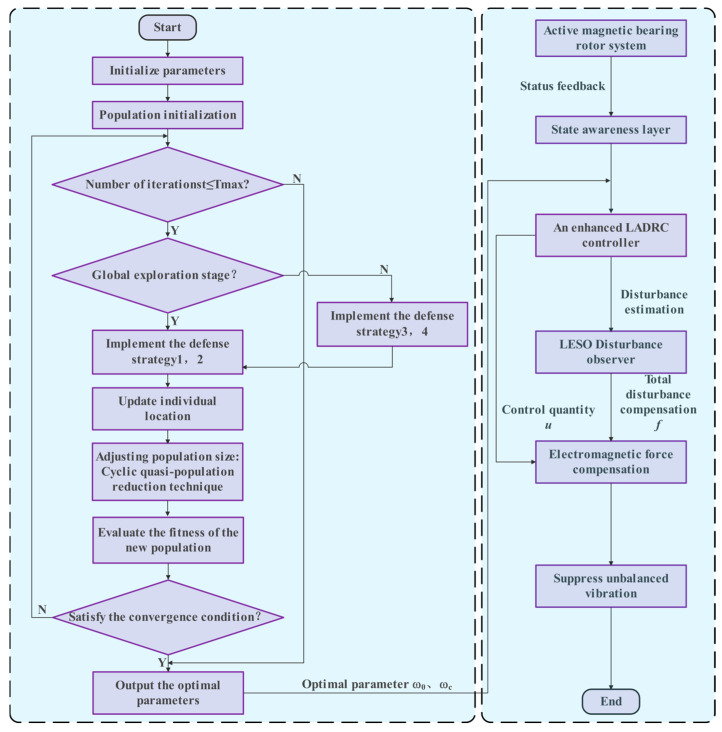
CPO-ELADRC algorithm process.

The workflow of the proposed CPO-ELADRC algorithm is structured into four main stages: initialization, parameter optimization, disturbance suppression, and iterative update. The detailed operational steps of the algorithm are given in Figure 8.

Step 1: Considering rotor unbalance vibration in magnetic bearing systems, an equivalent 4-DOF dynamic model of the rotor system is established to serve as the foundation for control strategy development.

Step 2: Real-time rotor states—including displacement, rotational speed, and control current—are acquired to construct a comprehensive state-space vector that reflects the system’s dynamic behavior.

Step 3: Sensor signals are integrated and processed to form the current system state vector, which serves as real-time input to the control algorithm.

Step 4: Initialize the parameters of the CPO algorithm, including population size, dimensionality, maximum number of iterations, and parameter boundaries. Simultaneously, set the initial values of the ELADRC controller parameters (e.g., $\omega_{0}$ and $\omega_{c}$).

Step 5: During the global exploration phase, the algorithm simulates the porcupine’s long-range threat perception by generating new candidate solutions using normally distributed random numbers (Equation (32)), thereby expanding the search space and avoiding local optima. The direction of parameter updates is adjusted based on the current best solution and differences among randomly selected individuals (Equation (33)) to enhance exploration diversity. In the local exploitation phase, fine-tuning is performed using the defense factor and odor diffusion factor (Equation (34)), enabling the algorithm to converge toward the optimal solution. To escape local optima, an inelastic collision force model (Equation (35)) is applied, improving convergence speed and global search ability.

Step 6: The fitness function evaluates each individual, and the porcupine population is ranked to select the best candidate in the current iteration. If this candidate surpasses the historical global best solution, the global optimum is updated. A convergence check is then performed: if the termination condition is not met, the population continues to evolve using the defined position update strategy; otherwise, the algorithm terminates and outputs the final optimized parameters.

Step 7: The $\left\{\omega_{0}^{*},\;\omega_{c}^{*}\right\}$ obtained from the CPO algorithm, is updated in real time to configure the LADRC structure.

Step 8: Based on the current system state vector and control input *u*, the LESO estimates the total system disturbance $\hat{f}$ in real time, enabling precise disturbance compensation.

Step 9: Using the *u* from the ELADRC controller and the $\hat{f}$, the required electromagnetic force compensation is calculated and transformed into a corresponding control current signal.

Step 10: The electromagnetic force compensation is combined with the $\hat{f}$ to actively cancel the total disturbance. This drives the magnetic bearing system to generate an electromagnetic force that is equal in magnitude and opposite in phase to the disturbance, effectively suppressing rotor vibrations caused by unbalance or external excitations and achieving robust vibration attenuation.

## 4. Simulation Experiments and Results Analysis

### 4.1. Simulation Parameter Settings

The simulation model was developed in MATLAB/Simulink R2022b, focusing on a 4-DOF magnetic bearing rotor system, and integrates four main functional modules: the mechanical dynamics module, the electromagnetic force linearization module, the CPO-ELADRC module, and the disturbance input module. Closed-loop control is realized through state feedback and dynamic parameter tuning. The architecture follows a hierarchical workflow of state sensing, parameter optimization, disturbance suppression, and actuation. The mechanical dynamics module is constructed based on the 4-DOF rotor dynamic equations, incorporating unbalanced mass excitation, gyroscopic effects, and inertial coupling. The electromagnetic force linearization module simplifies the inherently nonlinear magnetic force characteristics by establishing a linear relationship among electromagnetic force, control current, and rotor displacement using the *k_i_* and *k_s_*. The CPO-ELADRC module integrates a generalized LESO, the CPO for adaptive parameter tuning, and the LADRC feedback control structure to enable real-time estimation and compensation of total disturbances. The disturbance input module introduces both step and sinusoidal disturbances to evaluate the control strategy’s robustness and adaptability across a wide range of operating conditions. Key simulation parameters of the magnetic bearing system are listed in Table 2, and the rotor speed is varied from 0 to 45,000 rpm to validate the controller’s performance under dynamic and time-varying environments.

### 4.2. Results Analysis

#### 4.2.1. Multi-Condition Disturbance Rejection Performance

**Figure 9:**
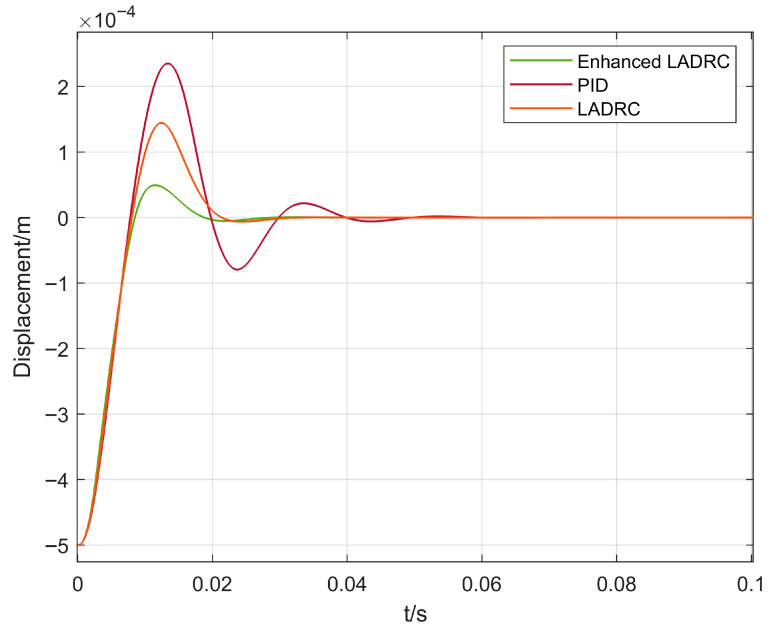
Displacement response curve under unit step excitation.

**Figure 10:**
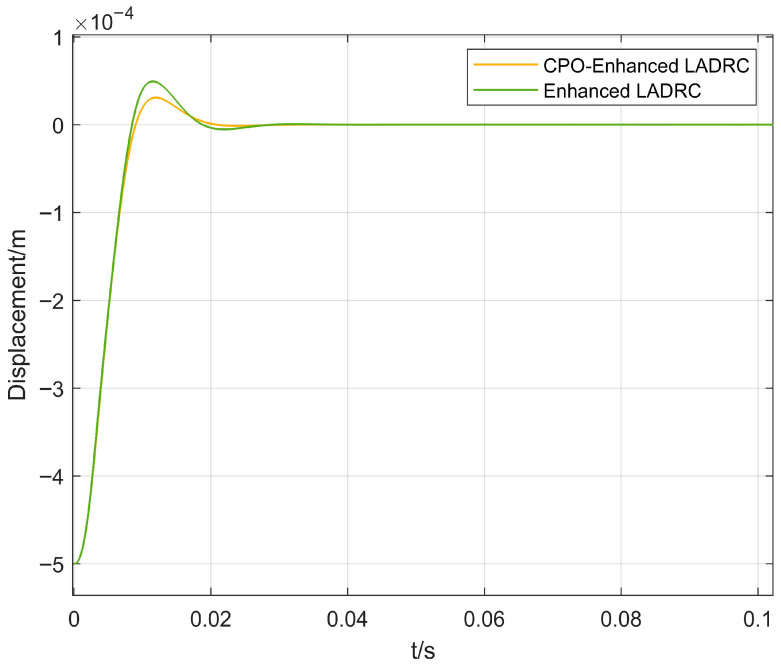
Displacement response curve under unit step excitation.

**Figure 11:**
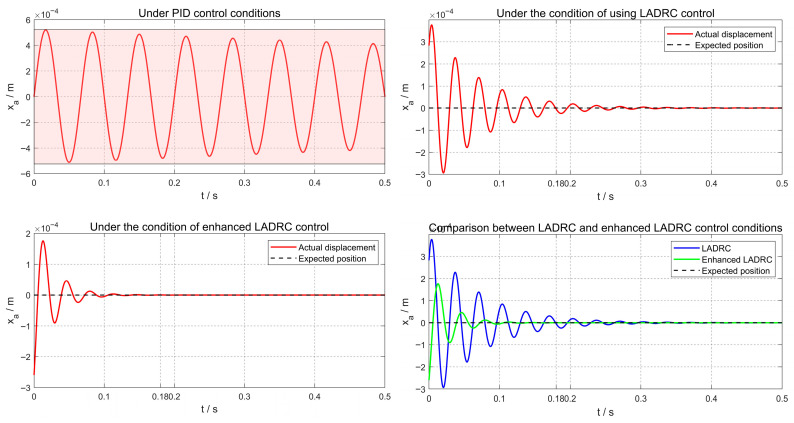
Displacement Response Curve under Sinusoidal Excitation.

**Figure 12:**
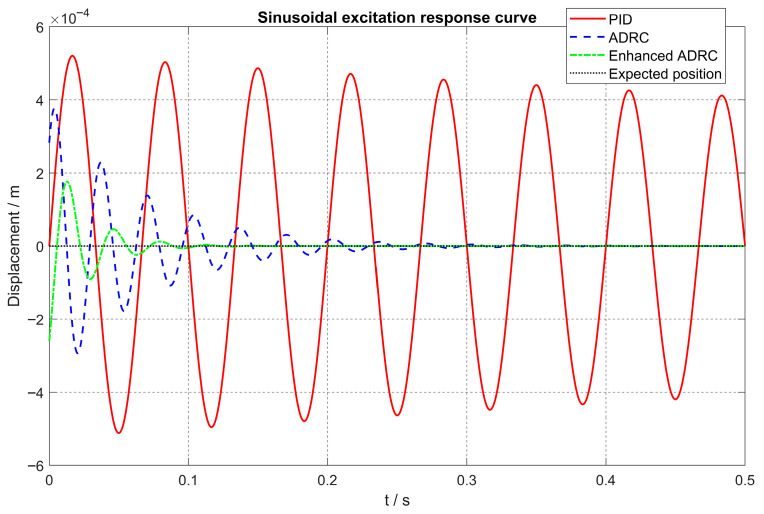
Comparison of Displacement Response Curves under Sinusoidal Excitation for Different Algorithms.

**Figure 13:**
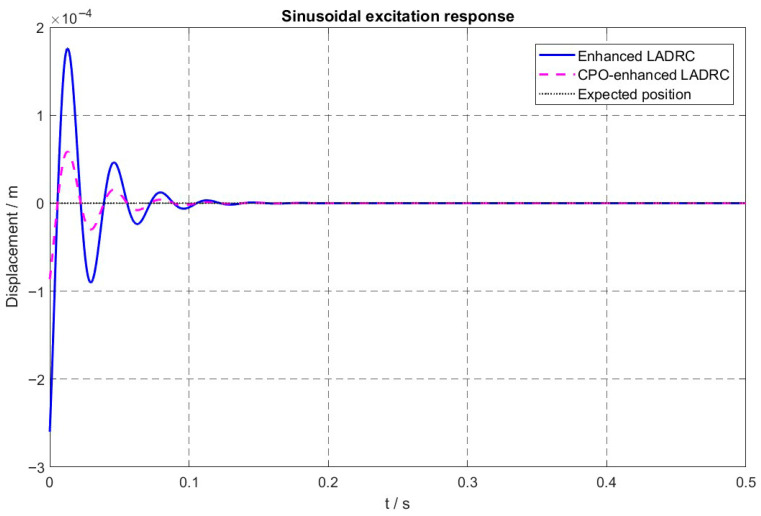
Comparison of Displacement Response Curves under Sinusoidal Excitation for Different Algorithms.

In Figure 9 and Figure 10, the dynamic response performance of the proposed CPO-ELADRC algorithm is compared with that of conventional PID, LADRC, and ELADRC algorithms under a unit step current disturbance. The simulation results indicate that the proposed strategy achieves a rotor displacement settling time of 0.019 s, representing performance improvements of 66.7%, 23.8%, and 22.5% over the PID, LADRC, and ELADRC methods, respectively. Additionally, the maximum displacement peak is reduced to 0.31 μm, corresponding to reductions of 86.8%, 78.5%, and 36.7% compared to the three baseline controllers. The steady-state displacement error is nearly 0, demonstrating the superior disturbance rejection capability and high control precision of the CPO-ELADRC approach. A detailed comparison of the quantitative performance indicators is presented in Table 3. While Figure 11, Figure 12 and Figure 13 present results at a representative low-frequency excitation, extensive simulations were conducted across the full speed range of 0–45,000 rpm. These simulations confirm that the proposed CPO-ELADRC controller maintains consistent disturbance rejection performance under varying rotational speeds, where speed-dependent effects such as gyroscopic coupling and parameter variations are effectively absorbed into the total disturbance estimated by the LESO.

Under step disturbance conditions, the CPO algorithm adaptively adjusts the LESO bandwidth and controller parameters in real time, enabling rapid disturbance estimation and compensation. This dynamic tuning mechanism effectively eliminates the response lag associated with insufficient bandwidth in traditional fixed-parameter designs and significantly enhances the system’s dynamic tracking performance.

In Figure 11, Figure 12 and Figure 13, the vibration suppression performance of the proposed CPO-ELADRC strategy was evaluated and compared with that of PID, LADRC, and ELADRC schemes under a unit-amplitude sinusoidal current disturbance. The simulation results show that the CPO-ELADRC algorithm reduced the rotor displacement regulation time to 0.094 s, representing improvements of 79.8%, 68.4%, and 14.5% over the PID, LADRC, and ELADRC controllers, respectively. Additionally, the maximum displacement peak was reduced to 0.64 $\times \;10^{-4}$ m, yielding performance enhancements of 87.7%, 83.0%, and 63.6%, respectively. The steady-state displacement error approached 0, confirming the superior vibration attenuation capability of the proposed method. The detailed quantitative performance metrics are summarized in Table 4, demonstrating that the CPO-ELADRC strategy meets the high-precision levitation requirements of advanced magnetic bearing systems.

Under sinusoidal disturbance conditions, the enhanced control strategy leverages the CPO algorithm to enable adaptive learning and compensation of time-varying disturbances, effectively mitigating phase lag at higher frequencies. This capability ensures accurate and timely disturbance rejection across a wide frequency spectrum, thereby avoiding the performance degradation typically observed in conventional ADRC approaches under complex and dynamic operating conditions.

#### 4.2.2. Analysis of Control Current Characteristics

**Figure 14:**
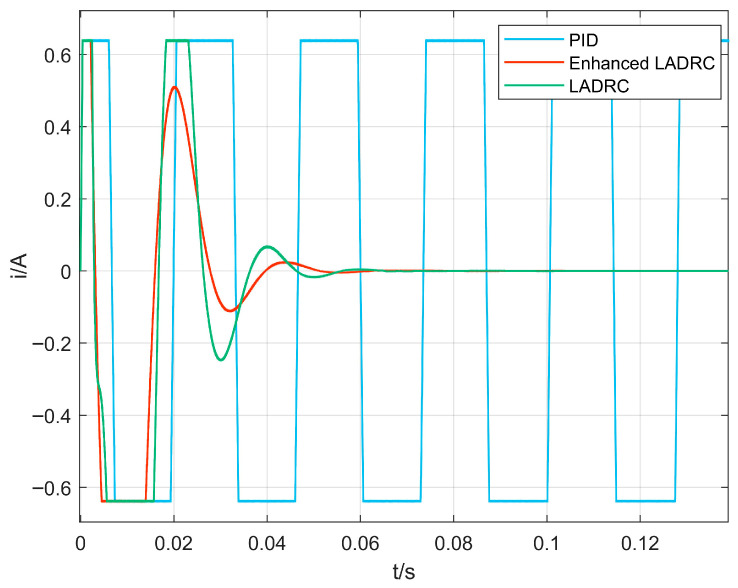
Comparison of control current profiles for different algorithms.

**Figure 15:**
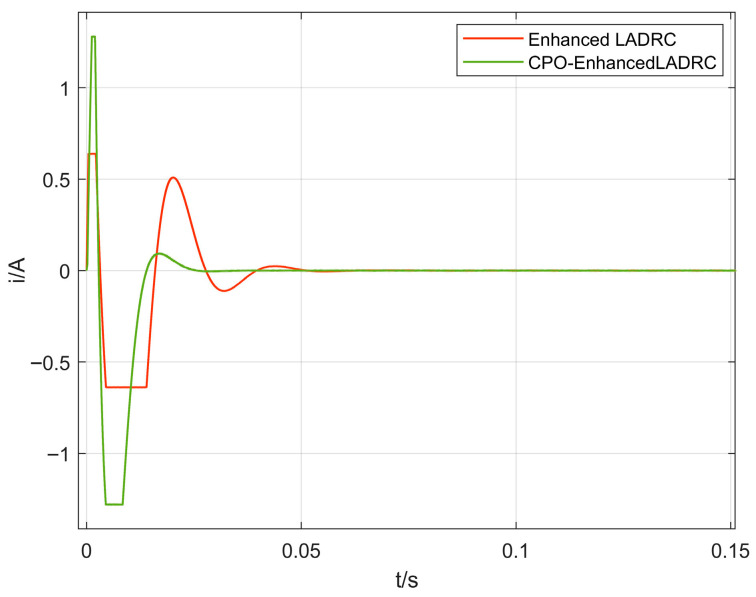
Comparison of control current profiles for different algorithms.

Figure 14 and Figure 15 illustrate the control current responses under various algorithms. With the CPO-ELADRC approach, the peak current is notably minimized to 0.087 A, reflecting substantial reductions of 86.4%, 86.1%, and 82.9% compared to PID, LADRC, and ELADRC controllers, respectively. Beyond reducing the peak value, the strategy also achieves visibly smoother current profiles with diminished fluctuation amplitudes. This outcome suggests that the adaptive tuning enabled by the CPO algorithm contributes not only to effective vibration attenuation but also to improved energy efficiency. Furthermore, by alleviating current surges and suppressing saturation risks, the method enhances the operational stability and long-term dependability of the magnetic bearing system.

## 5. Experimental Validation

**Figure 16:**
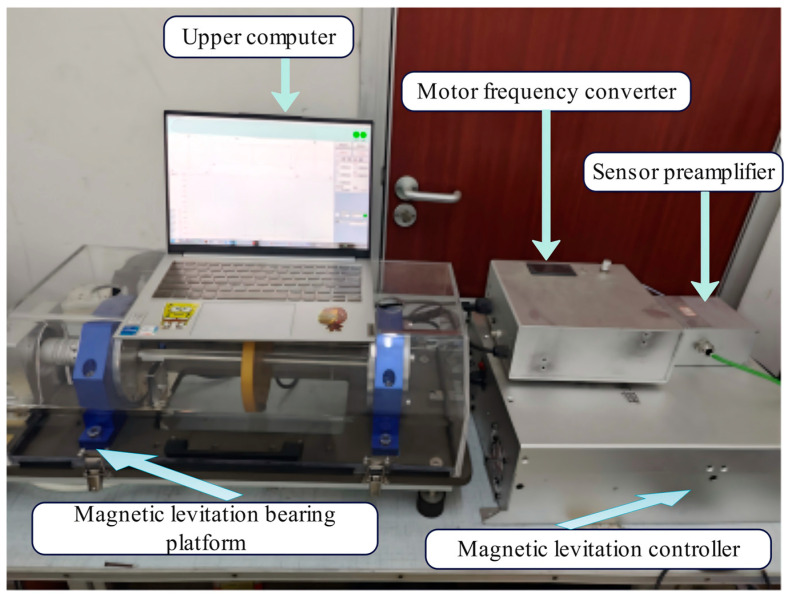
Magnetic levitation motor rotor system test bench.

To assess the practical applicability of the proposed CPO-ELADRC approach, a hardware-in-the-loop experimental system was constructed using a dedicated magnetic bearing testbed. The system’s control performance was examined under both step and sinusoidal disturbance conditions, as well as across a broad range of rotational speeds. In Figure 16, the experimental platform integrates a magnetic suspension rotor assembly, high-resolution sensor units, real-time control modules, and a data acquisition subsystem. Detailed specifications of the key hardware components involved in the setup are summarized in Table 5.

The experimental magnetic levitation platform is configured with radial magnetic bearings offering 4-DOF in the *x*- and *y*-directions at both the *a* and *b* ends, complemented by backup bearings and driven by a 2.2 kW permanent magnet synchronous motor. The rotor features a total mass of 70 kg and an axial length of 0.822 m, with corresponding moments of inertia of *J*_x_ = *J*_y_ = 15.8 kg·m^2^ and *J*_z_ = 0.056 kg·m^2^. Displacement measurements are obtained using CWY-DO-20XLQ05-80Z4 eddy-current sensors, which provide a sensitivity of 10 V/mm and operate within a ±0.2 mm linear range. These sensors are positioned along the x and y axes at both bearing ends, sampling at a frequency of 10 kHz. The control core is built around the TI TMS320F28379D digital signal processor (DSP), running with a 100 μs control cycle and embedding both the CPO optimization algorithm and the LADRC control structure. Power amplification is handled via an H-bridge circuit, capable of delivering ±5 A with a control bandwidth of up to 20 kHz.

**Figure 17:**
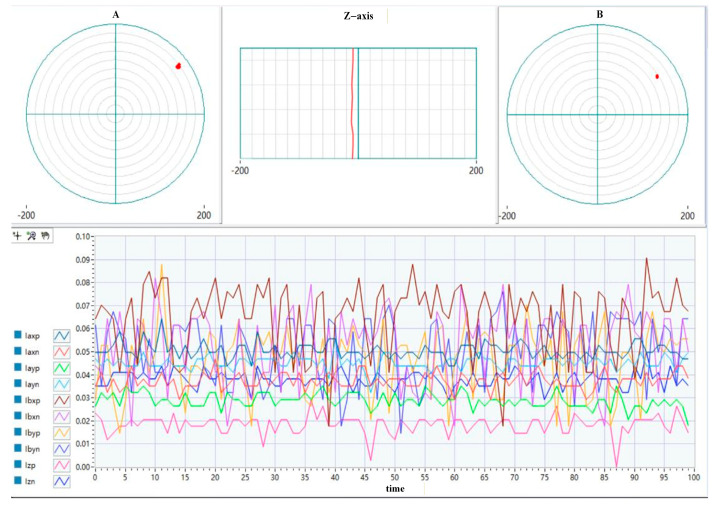
No CPO was added—ELADRC strategy floating display interface.

**Figure 18:**
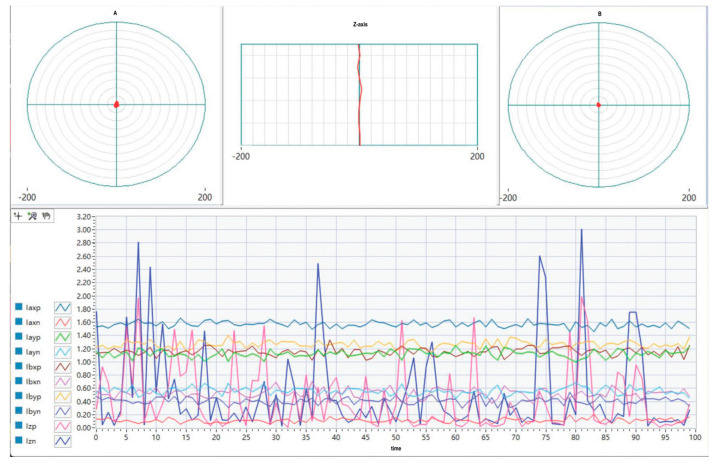
Add CPO- Improve the floating display interface of LADRC control strategy.

After the rotor was stably levitated at its equilibrium position, an acceleration experiment was performed in which the rotational speed was continuously increased from 0 to 45,000 rpm. The corresponding vibration responses of the rotor were recorded and analyzed. Figure 17 and Figure 18 present a comparative evaluation of the magnetic bearing system’s levitation interface performance with and without the integration of the CPO-ELADRC scheme. The comparison is conducted with respect to key metrics such as displacement accuracy, dynamic response stability, and disturbance rejection effectiveness. These results highlight the improvements in system behavior enabled by the proposed strategy and underscore its contribution to enhanced control precision and robustness.

In the absence of the CPO-ELADRC suppression strategy, the displacement response curves exhibit multicolored traces characterized by low-frequency periodic oscillations and distinct spikes under step disturbances. For example, when subjected to a 10% current disturbance, the displacement peak reaches 1.47 μm, with the system taking approximately 0.042 s to return to steady state. In contrast, the implementation of the CPO-ELADRC strategy results in smooth, high-frequency response curves with no visible overshoot. The fluctuation amplitude remains below 0.5 μm, and under sinusoidal disturbances, the total harmonic distortion (THD) is limited to 2.3%, indicating minimal harmonic content in the current. Upon a step disturbance, the system settles rapidly within 0.021 s without any overshoot. In Table 6, the CPO algorithm enables adaptive tuning of the LESO bandwidth, adjusting $\omega_{0}$ dynamically between 1500 rad/s and 2000 rad/s, which improves the accuracy of disturbance estimation by approximately 40%. Overall, experimental results confirm that this strategy enhances the suspension system’s positioning precision by 79.8% and improves dynamic response characteristics—halving the adjustment time—thus demonstrating the practical effectiveness of the proposed CPO-ELADRC scheme in active magnetic suspension applications.

## 6. Conclusions

The CPO-ELADRC strategy introduced in this work offers an effective solution to unbalanced vibrations and strong multiple-DOF coupling challenges in magnetic bearing systems. By leveraging a LESO for high-precision disturbance estimation and employing the CPO algorithm for dynamic parameter tuning, the proposed method enhances both control accuracy and adaptability. A comprehensive 4-DOF dynamic model of the magnetic bearing rotor is established, along with a linearized electromagnetic force model. Building upon this foundation, a disturbance observation and compensation mechanism is designed, while the CPO modeled after the crested porcupine’s multimodal defense behavior—optimizes key control parameters such as LESO bandwidth and LADRC gains. A state-space control structure is formulated by integrating rotor displacement, speed, and current, and a reward function balancing vibration suppression and energy efficiency enables multi-objective optimization through adaptive parameter tuning. The effectiveness of the strategy is verified through both MATLAB/Simulink simulations and physical experiments conducted on a magnetic levitation test platform. Results confirm that, across a broad range of rotational speeds, the proposed approach delivers superior performance in terms of vibration attenuation, dynamic response time, and energy consumption when compared to traditional PID, LADRC, and ELADRC controllers. Nevertheless, the proposed approach still has limitations. The CPO-based optimization increases computational complexity and may affect real-time performance. Extension to higher-degree-of-freedom systems could enlarge the optimization space, and LESO performance may be influenced by measurement noise. Future work will focus on improving computational efficiency, noise robustness, and scalability.

## Figures and Tables

**Table 1:** Selection of Optimization Parameters.

Parameter Category	Specific Parameter	Physical Meaning	Value Range	Optimization Objective
LESO Parameter	*ω*_0_	Determines disturbance estimation accuracy and noise rejection capability	[500, 2000] rad/s	Improve real-time estimation of high-frequency disturbances
LADRC Controller Parameter	*ω_c_*	Corresponds to PD feedback gain $k_{p}=\omega_{c}^{2},\;k_{d}=2\xi \omega_{c}\;(\xi =0.707)$	[300, 1500] rad/s	Balance system response speed and control energy consumption

**Table 2:** Parameters of the 4-DOF AMB Simulation Model.

Parameter	Description	Value
m	Rotor mass	15 kg
x	Rotor radial length in *x*-direction	0.04 m
y	Rotor radial length in *y*-direction	0.04 m
z	Rotor axial length (*z*-direction)	0.822 m
K*_i_*	Magnetic bearing current stiffness	250 N/A
K_s_	Magnetic bearing displacement stiffness	−1.2 × 107 N/m
*J_x_*	Rotor moment of inertia about *x*-axis	15.8 kg·m^2^
*J_y_*	Rotor moment of inertia about *y*-axis	15.8 kg·m^2^
*J_z_*	Rotor moment of inertia about *z*-axis	0.056 kg·m^2^

**Table 3:** Performance indices under unit step response.

Control Algorithm	Time (s)		$\mathrm{Displacement}\;(\times 10^{-4}$ m)	
	Peak Time	Settling Time	Peak Displacement	Steady-State Displacement
PID	0.013	0.070	2.35	0
LADRC	0.013	0.042	1.44	0
ELADRC	0.012	0.040	0.49	0
CPO-ELADRC	0.012	0.031	0.31	0

**Table 4:** Performance indices under unit sinusoidal excitation.

Control Algorithm	Time (s)		$\mathrm{Displacement}\;(\times 10^{-4}$ m)	
	Peak Time	Settling Time	Peak Displacement	Steady-State Displacement
PID	0.017	0.483	5.21	4.12
LADRC	0.0035	0.301	3.77	0
ELADRC	0.013	0.123	1.76	0
CPO-ELADRC	0.013	0.107	0.64	0

**Table 5:** Core Parameters of the Experimental System.

Parameter Category	Specific Parameter	Value
Rotor Parameters	Mass	15 kg
	Axial Length	0.822 m
	Moment of Inertia (x/y)	15.8 kg·m^2^
Sensor Parameters	Sensitivity	10 V/mm
	Sampling Frequency	10 kHz
Controller Parameters	Core Chip	TMS320F28379D
	Control Period	100 μs
Motor Parameters	Rated Power	2.2 kW
	Speed Range	0–45,000 rpm

**Table 6:** Displacement value and positioning accuracy.

Metric	Without CPO-ELADRC	With CPO-ELADRC
Radial displacement (X/Y axes)	1.47~2.41 μm	0.295~4.935 μm
Axial displacement (Z axis)	−1.7~−2.0 μm	−1.764 μm
Positioning stability	±0.5 μm	±0.1 μm

## Data Availability

The original contributions presented in this study are included in the article. Further inquiries can be directed to the corresponding author.
